# Supplementary material for: Altered B cell activation contributes to the immunopathogenesis of childhood arthritis-associated uveitis
Source: Nat Commun. 2026 Feb 3;17:714. doi: 10.1038/s41467-025-68264-5 (PMC12868682; doi:10.1038/s41467-025-68264-5)
Supplement: Supplementary file 1 — Supplementary Information [file 41467_2025_68264_MOESM1_ESM.pdf]

Title: Altered B cell activation contributes to the immunopathogenesis of childhood arthritis-associated uveitis.

1st author: Dr Bethany R Jebson (b.jebson@ucl.ac.uk)

Corresponding author: Dr Elizabeth C. Rosser (e.rosser@ucl.ac.uk)

Supplementary Materials.

This PDF file includes:

Figures S1 to S11

Table S1 to S10

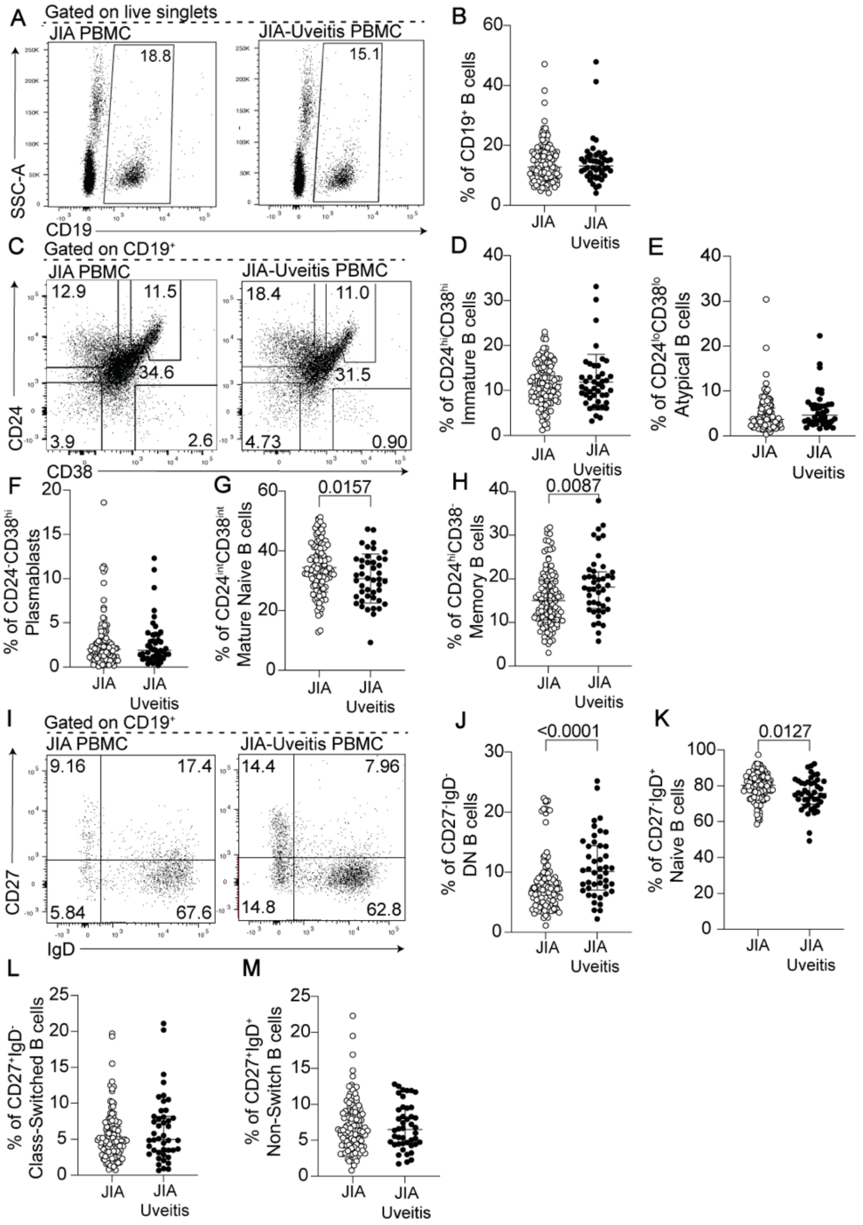

Supplementary Figure 1. B cell subset frequency within peripheral blood mononuclear cells of JIA patients with uveitis is altered compared to JIA patients with no uveitis. All data is generated from peripheral blood mononuclear cells (PBMC) collected from JIA patients with no uveitis (JIA, *n*=116) and JIA patients with uveitis (JIA-Uveitis, *n*=44). **A.** Representative flow cytometry plots and **B.** dot plot showing the frequency of $CD19^+$ B cells within live singlets. **C.** Representative flow cytometry plots and dot plots showing the frequency of **D.** $CD24^{hi}CD38^{hi}$ Immature B cells, **E.** $CD24^{lo}CD38^{lo}$ Atypical Memory B cells, **F.** $CD24^-CD38^{hi}$ Plasmablasts ,**G.** $CD24^{int}CD38^{int}$ Mature Naive B cells, **H.** $CD24^{hi}CD38^-$ Memory B cells within $CD19^+$ live singlets. **I.** Representative flow cytometry plots showing the frequency of **J**. $CD27^-IgD^-$ Double Negative (DN) B cells **K.** $CD27^-IgD^+$ Naïve B cells, **L.** $CD27^+IgD^-$ Class-Switched B cells, **M.** $CD27^+IgD^+$ Non-Switch B cells within $CD19^+$ live singlets. Significance of difference between groups was determined using the two-tailed Mann-Whitney test. P values below or equal to 0.05 are shown on graphs but the significance threshold was set at ≤0.005 to adjust for multiple testing (Bonferroni). Error bars represent median ± IQR for groups.

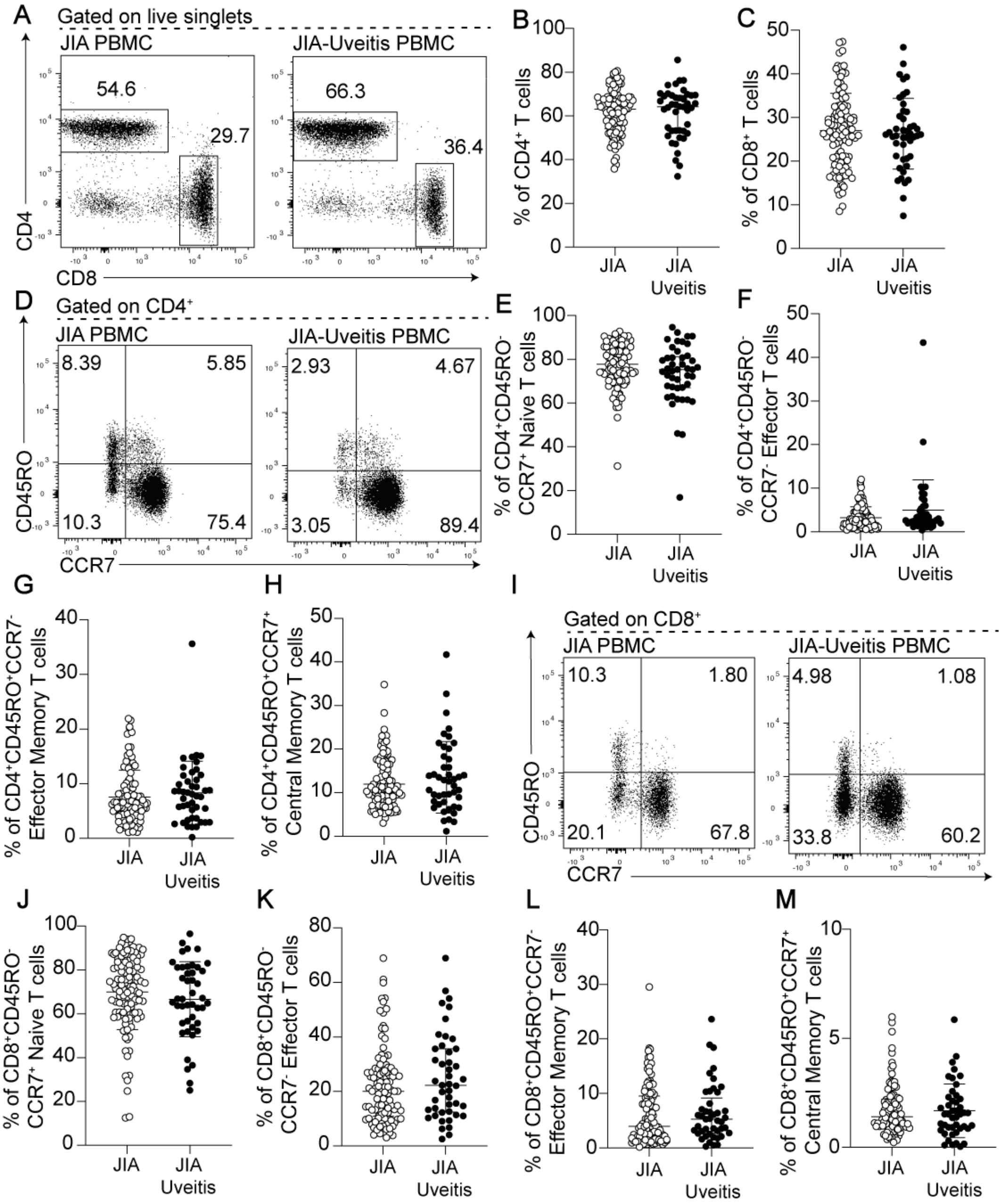

Supplementary Figure 2. Similar frequencies of naïve and memory CD4+ T cells and CD8+ T cells within peripheral blood mononuclear cells are observed between JIA patients with and without uveitis. All data is generated from PBMC collected from JIA patients with no uveitis (JIA, *n*=107) and JIA patients with uveitis (JIA-Uveitis, *n*=45). **A.** Representative flow cytometry and dot plots showing the frequency of **B.** $CD4^+$ T cells and **C.** $CD8^+$ T cells within $CD3^+$ live singlets. **D.** Representative flow cytometry plots and dot plots showing **E.** $CD45RO^-CCR7^+$ Naïve T cells, **F.** $CD45RO^-CCR7^-$ Effector T cells, **G.** $CD45RO^+CCR7^-$ Effector Memory T cells and **H.** $CD45RO^+CCR7^+$ Central Memory T cells within the $CD4^+$ live singlets. **I.** Representative flow cytometry plots and dot plots showing **J.** $CD45RO^-CCR7^+$ Naïve T cells, **K.** $CD45RO^-CCR7^-$ Effector T cells, **L.** $CD45RO^+CCR7^-$ Effector Memory T cells and **M.** $CD45RO^+CCR7^+$ Central Memory T cells within the $CD8^+$ live singlets. Significance of difference between groups was determined using the two-tailed Mann-Whitney test. P values below or equal to 0.05 are shown on graphs but the significance threshold was set at ≤0.005 for CD4 populations and ≤0.0125 for CD8 populations to adjust for multiple testing (Bonferroni). Error bars represent median ± IQR for groups.

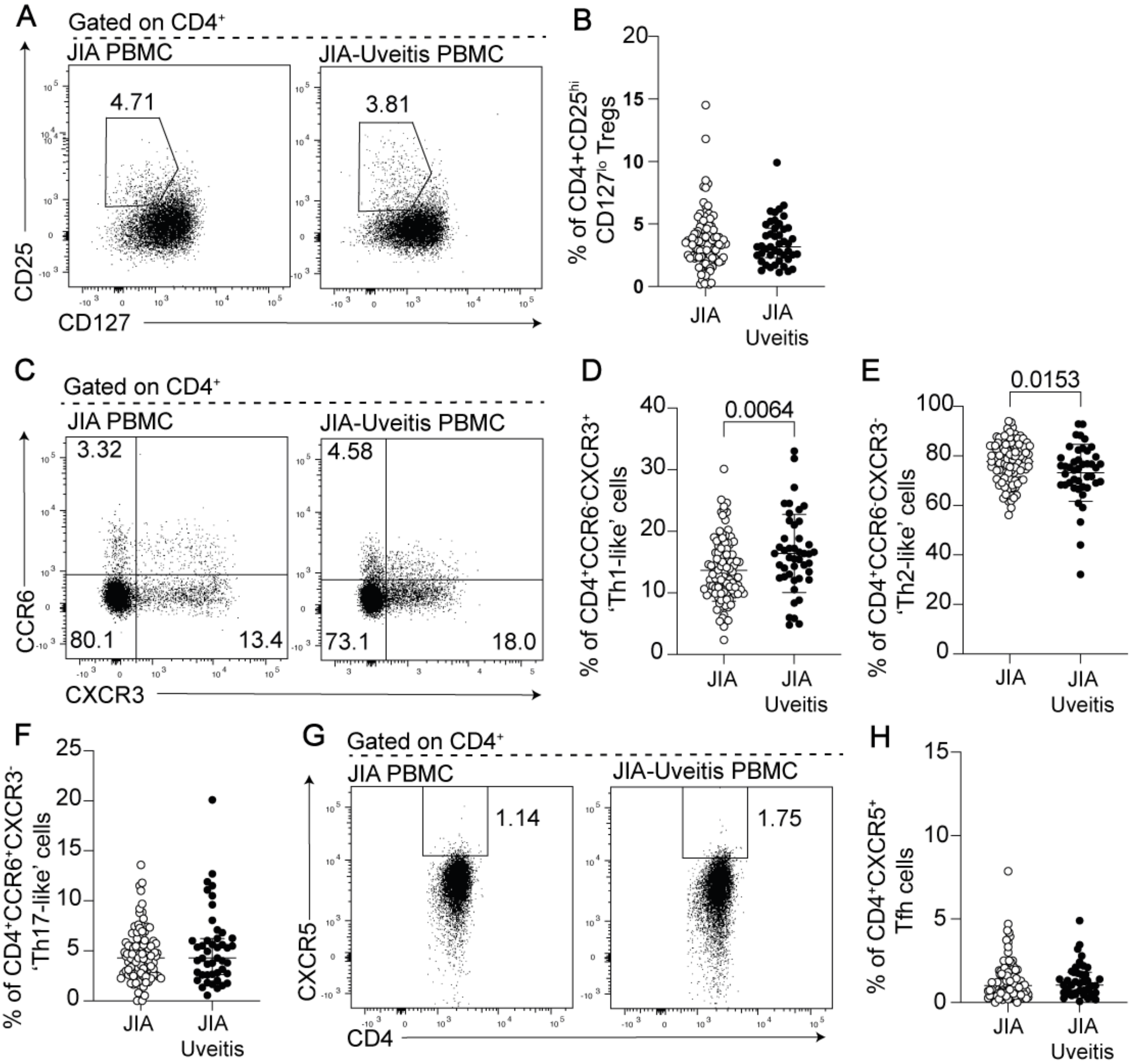

Supplementary Figure 3. T helper cell subsets within peripheral blood mononuclear cells are altered in JIA patients with uveitis compared to JIA patients without uveitis. All data were generated from PBMC collected from JIA patients with no uveitis (JIA, *n*=107) and JIA patients with uveitis (JIA-Uveitis, *n*=45), unless otherwise stated. **A.** Representative flow cytometry plots and **B.** dot plot showing the frequency of $CD25^{hi}CD127^{lo}$ T regulatory (Treg) cells within $CD4^{+}$ live singlets. **C.** Representative flow cytometry plots and dot plots showing the frequency of **D.** $CCR6^{-}CXCR3^{+}$ 'Th1 like' T cells, **E.** $CCR6^{-}CXCR3^{-}$ 'Th2 like' T cells and **F.** $CCR6^{+}CXCR3^{-}$ 'Th17 like' T cells within the $CD4^{+}$ live singlets. **G.** Representative flow cytometry plots showing the frequency of **H.** $CXCR5^{+}$ T follicular helper (Tfh) T cells within $CD4^{+}$ live singlets from JIA patients with no uveitis (JIA, n=101) and JIA with uveitis (JIA-Uveitis, n=42). Significance of difference between groups was determined using two-tailed unpaired T-test for normally distributed groups (**D**), and two-tailed Mann-Whitney test where groups were non-normally distributed **(B,E,F,H)**. P values below or equal to 0.05 are shown on graphs but the significance threshold was set at ≤0.005 to adjust for multiple testing (Bonferroni). Error bars represent mean ± SD for normally distributed groups and median ± IQR for non-normal groups.

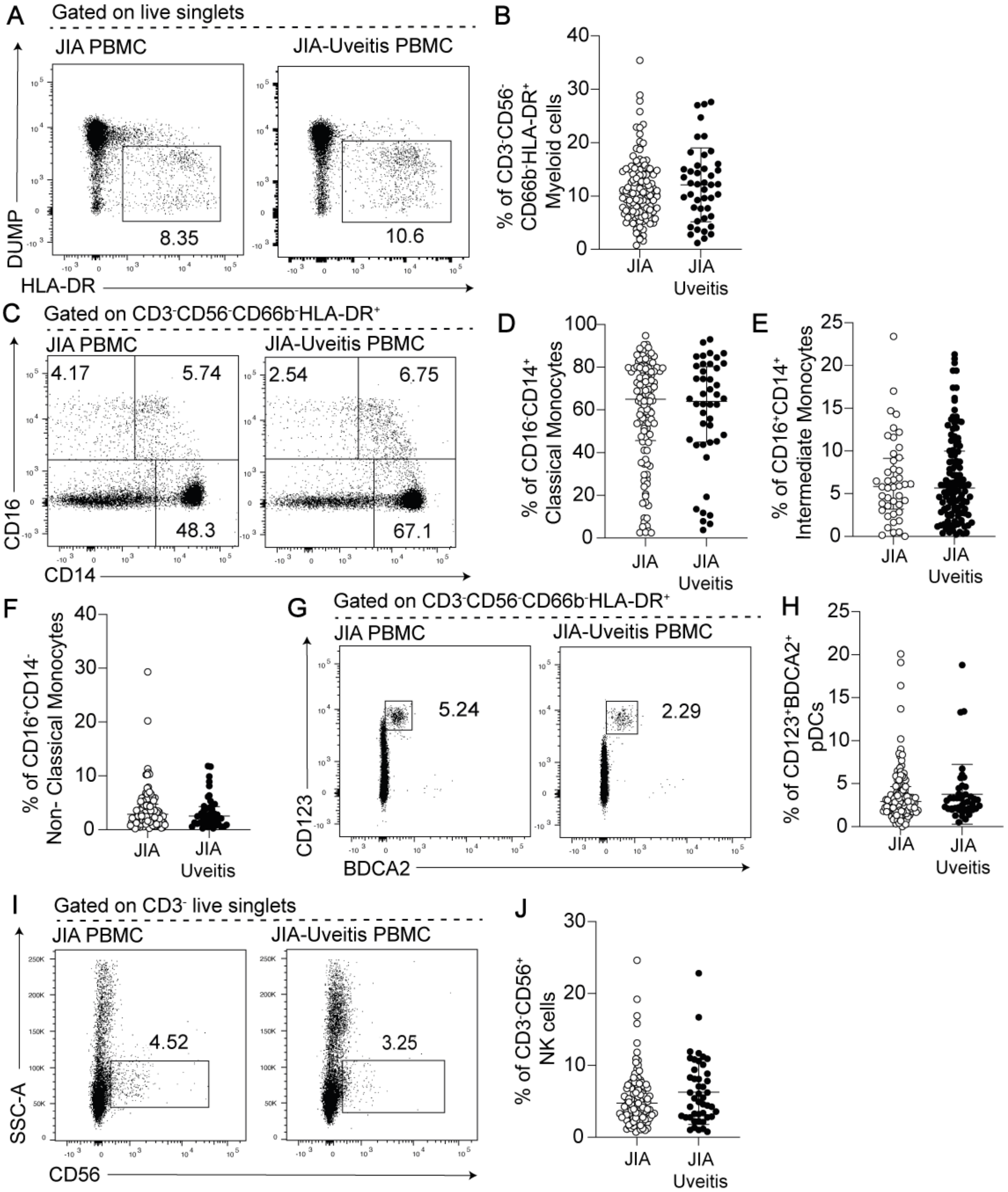

Supplementary Figure 4. Similar frequencies of Innate immune cell populations within peripheral blood mononuclear cells are observed between JIA patients with and without uveitis. All data were generated from PBMC collected from JIA patients with no uveitis (JIA, *n*=116) and JIA patients with uveitis (JIA-Uveitis, *n*=44). **A.** Representative flow cytometry plots and **B.** dot plot showing the frequency of CD3$^-$CD56$^-$CD66b$^-$ (DUMP) HLA-DR$^+$ Myeloid cells within live singlets. **C.** Representative flow cytometry and dot plots showing **D.** CD16$^-$CD14$^+$ Classical Monocytes, **E.** CD16$^+$CD14$^+$ Intermediate Monocytes and **F.** CD16$^+$CD14$^-$ Non-Classical Monocytes within HLA-DR$^+$ live singlets. **G.** Representative flow cytometry and **H.** dot plot showing the frequency of CD123$^+$BDCA2$^+$ plasmacytoid dendritic cells (pDCs) within CD3$^-$CD56$^-$CD66b$^-$ (DUMP) HLA-DR$^+$ Myeloid cells. **I.** Representative flow cytometry plots and **J.** dot plot showing the frequency of CD56$^+$ Natural Killer (NK) cells within live singlets. Significance of difference between all groups was determined using the two-tailed Mann-Whitney test. P values below or equal to 0.05 are shown on graphs but the significance threshold was set at ≤0.01 for myeloid populations and ≤0.05 for NK cells to adjust for multiple testing (Bonferroni). Error bars represent median ± IQR.

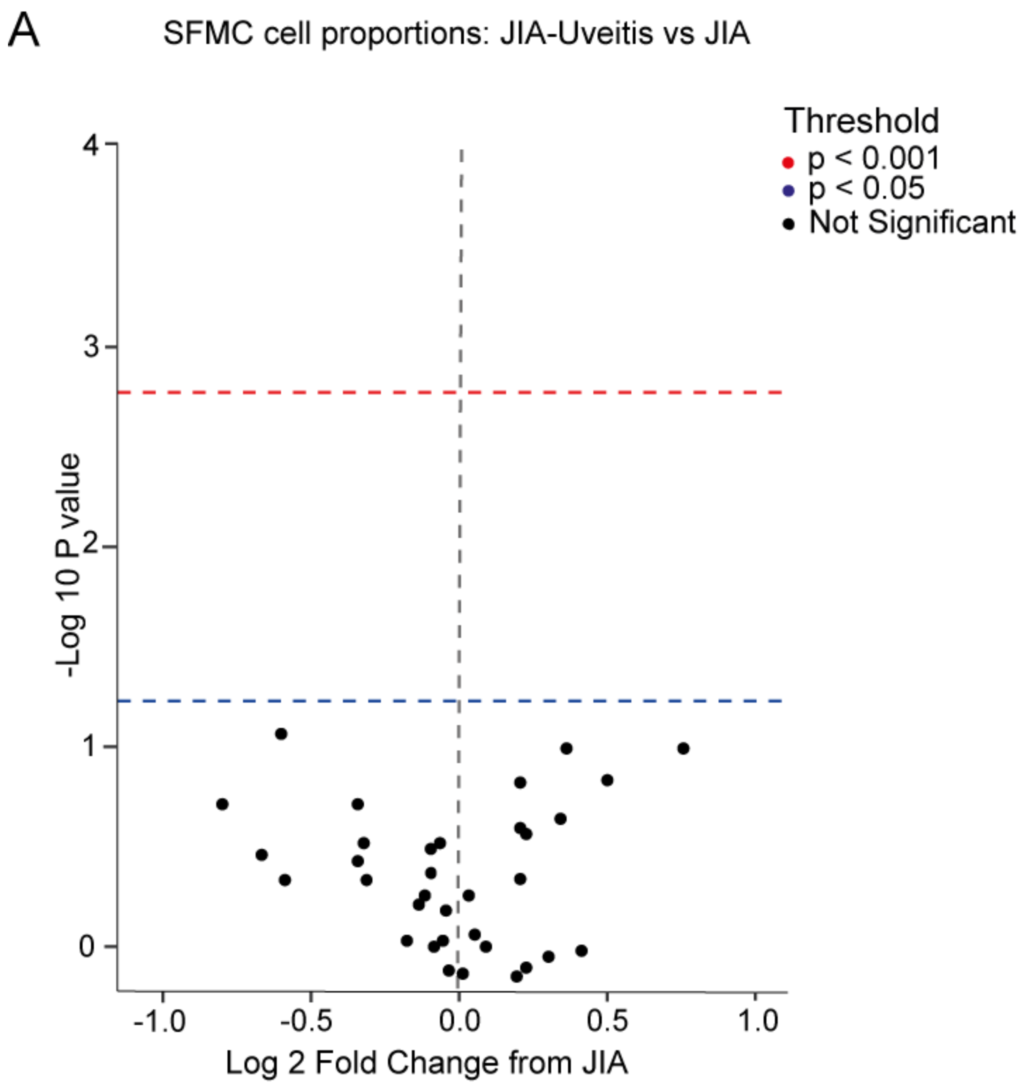

Supplementary Figure 5. There are no significant differences in immune cell populations within the synovial fluid of JIA patients with uveitis (JIA-Uveitis) and JIA patients with arthritis alone (JIA). All data are generated from SFMC collected from JIA patients with no uveitis (JIA, *n*=30) and JIA patients with uveitis (JIA-Uveitis, *n*=18). **A.** Volcano plot showing all 34 measured cell populations within the SFMC of JIA-Uveitis patients. Significance thresholds were set at $p \leq 0.05$ (blue dashed line) and $p \leq 0.0015$ (red dashed line; Bonferroni-corrected threshold, 0.05/34 populations).

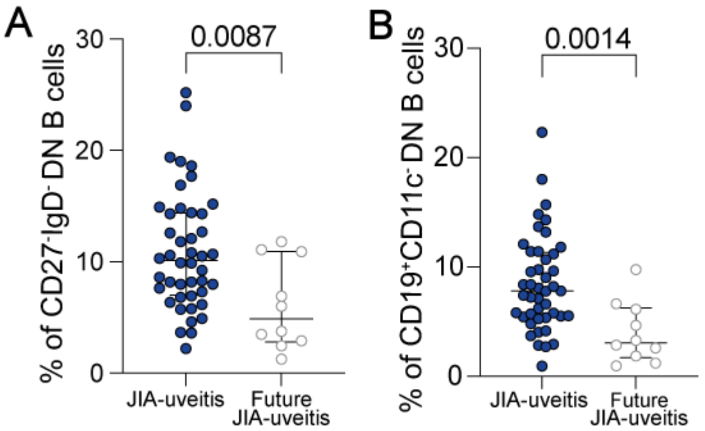

Supplementary Figure 6. Dot plot showing the proportion of DN and CD11c- DN B cells CD19+ B cells in the peripheral blood (PB) of JIA patients with uveitis (*n*=44), and patients who went on to develop uveitis after the time of sample (Future JIA-uveitis, *n*=10). Error bars represent median ± IQR. Significance of difference between all groups was determined using the two-tailed Mann-Whitney test. P-values ≤ 0.05 are shown on graphs.

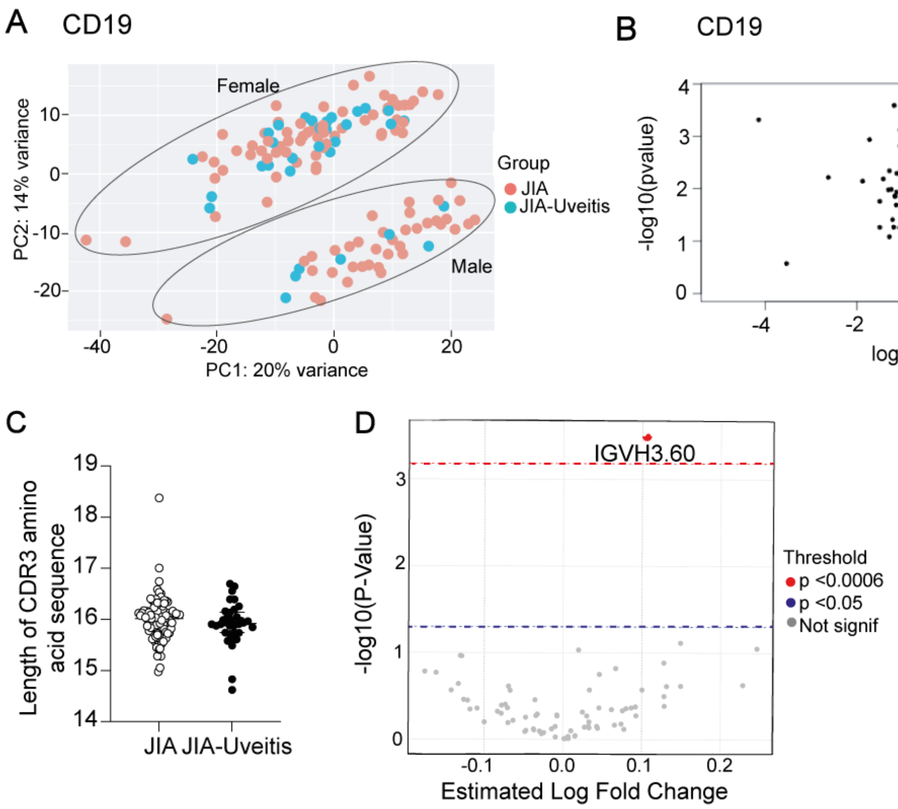

Supplementary Figure 7. Principal component analysis (PCA) and volcano plot of differentially abundant transcripts in CD19+ B cells within the peripheral blood of JIA and JIA-uveitis patients. PCA analysis utilising the top 500 genes expressed in each cell population, **A.** PCA of CD19+ B cells in the PBMC of JIA with no uveitis (JIA, *n*=101) and JIA-Uveitis patients (JIA-Uveitis, *n*=33). **B.** Volcano plot of $CD19^+$ B cells. Differentially expressed genes significance threshold of log2FoldChange greater than 1 and p-adjusted ≤0.05. Following data is based on B Cell Receptor repertoire RNA-seq data from CD19+ B cells (JIA *n*=100, JIA-Uveitis *n*=33). **C.** Average CDR3 amino acid length. The significance of difference between groups was determined using the two-tailed Mann-Whitney test. P values have been adjusted for sex and age. Error bars represent median ± IQR for groups. **D.** Volcano plot showing all 76 measured IGH genes within the BCR repertoire analysis. Significance thresholds for differential abundance were set at $p \leq 0.05$ (uncorrected) and $p \leq 0.0006$ (Bonferroni corrected). Error bars represent median ± IQR.

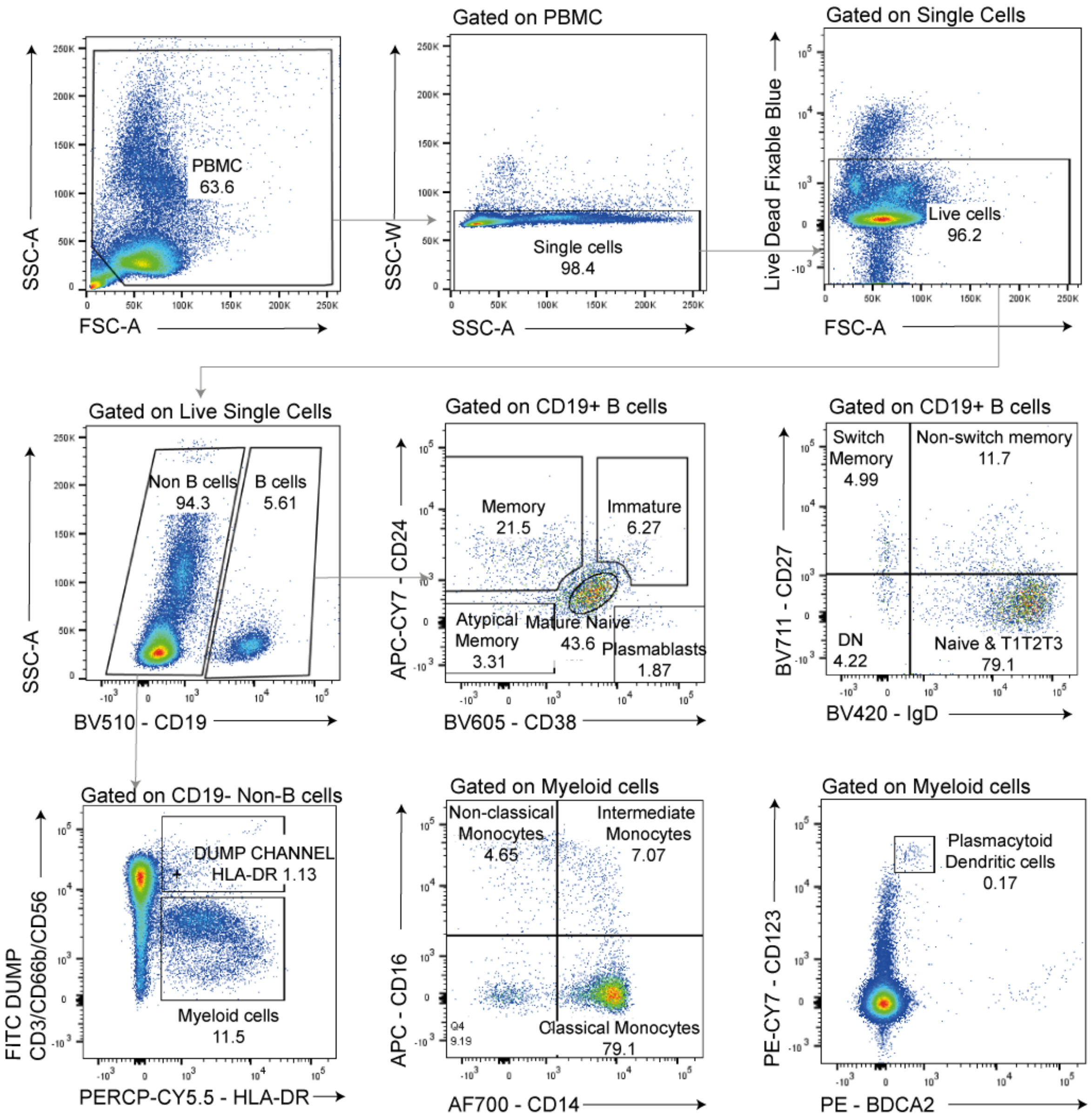

Supplementary figure 8. Flow cytometry gating strategies showing identification of canonical B cell subsets and myeloid subsets within human peripheral blood mononuclear cells.

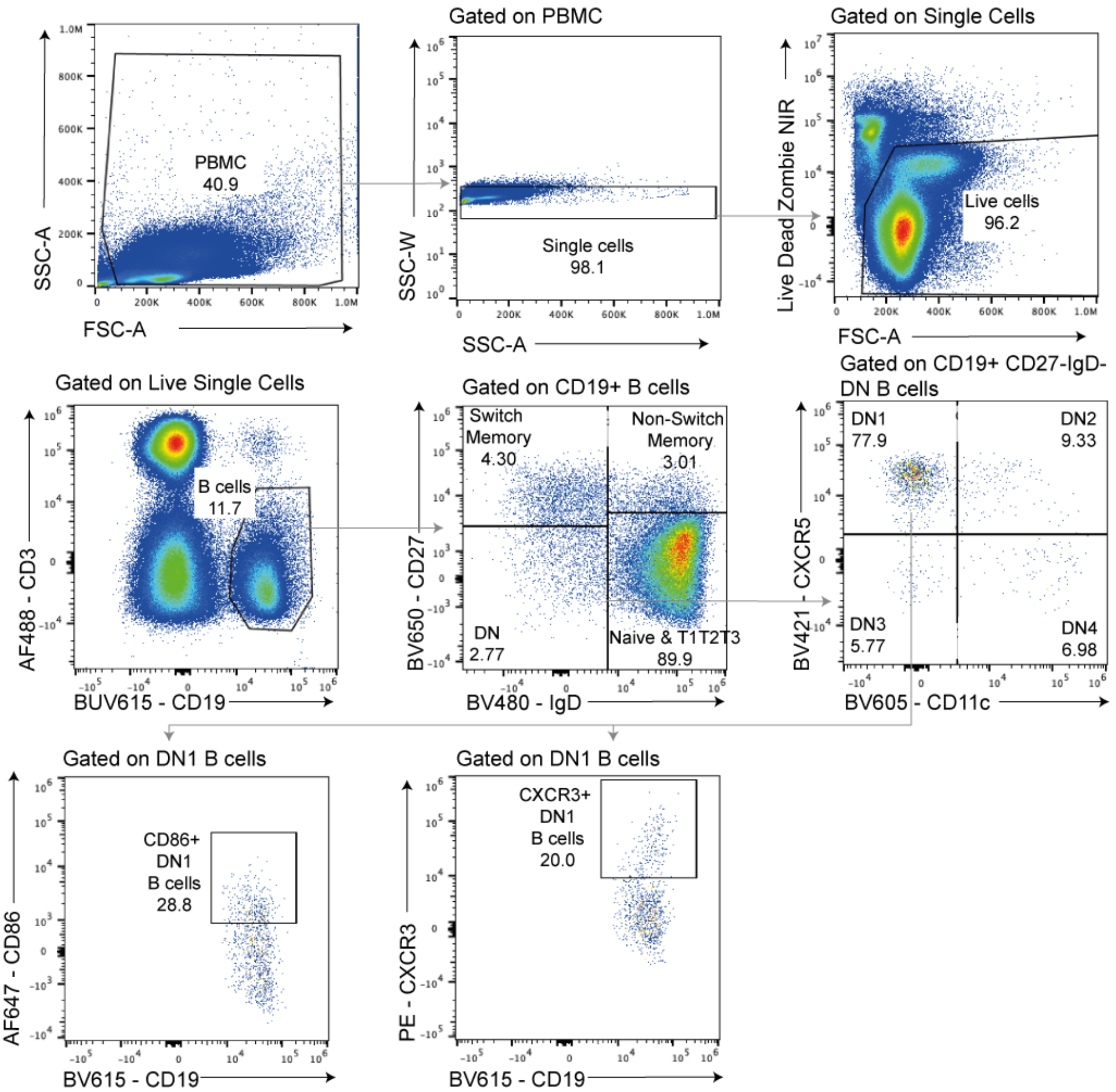

Supplementary figure 9. Flow cytometry gating strategies showing identification of human double negative (DN) B cell subsets within human peripheral blood mononuclear cells.

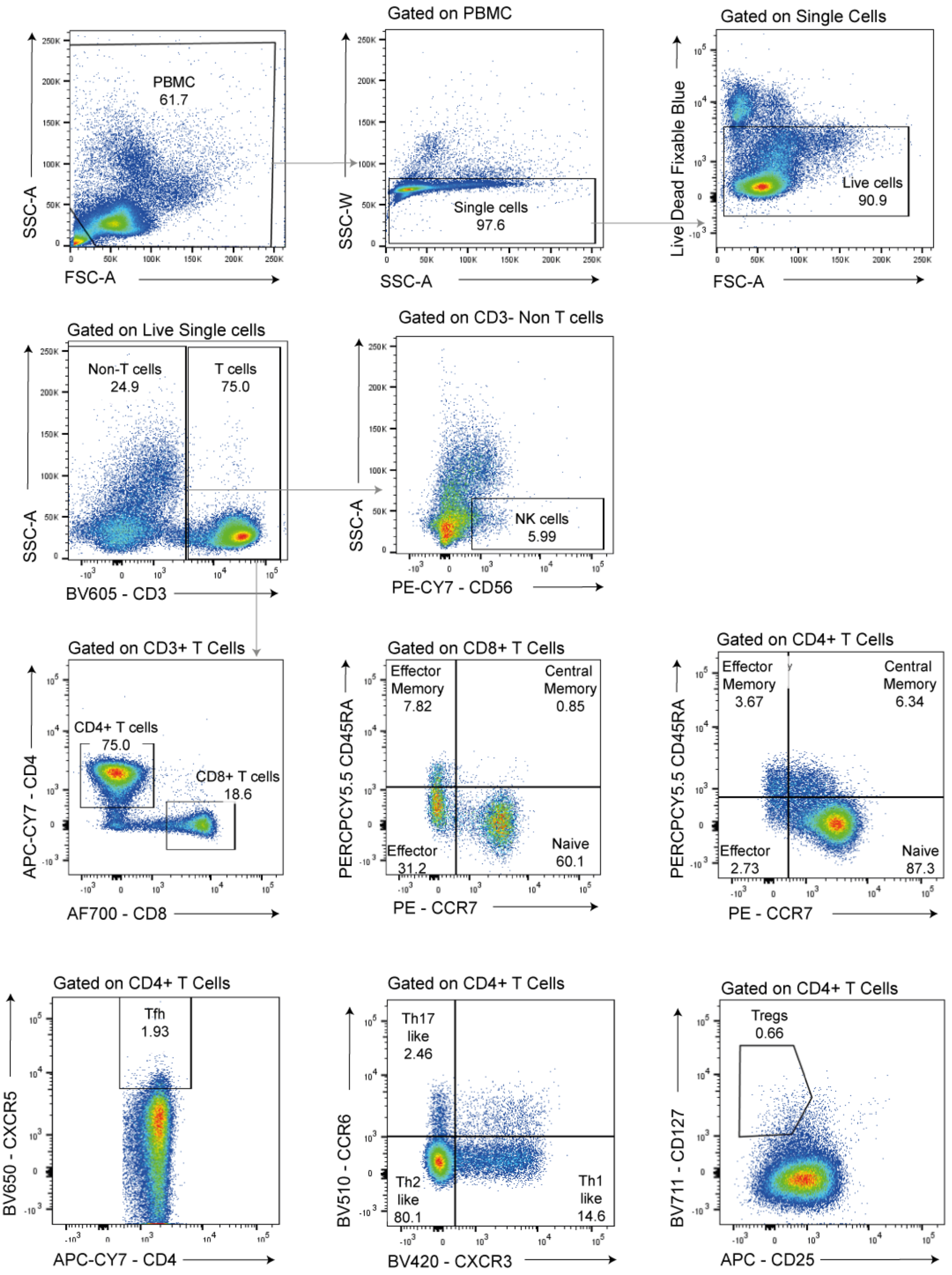

Supplementary figure 10. Flow cytometry gating strategies showing identification of T cells and NK cell subsets within human peripheral blood mononuclear cells.

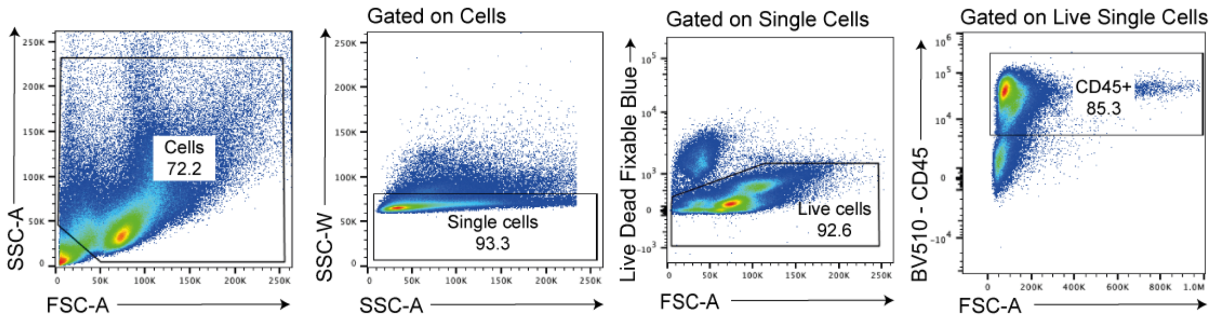

Supplementary figure 11. Flow cytometry gating strategy showing identification of lymphocytes within different mouse organs, prior to further analyses.

Supplementary Table 1. 34 included human immune cell populations with marker definitions.

Population	Definition
T cells	CD3+
CD4+ T cells	CD3+ CD4+
CD8+ T cells	CD3+ CD8+
NK cells	CD3- CD56+
Effector CD8 T cells	CD3+ CD8+ CCR7- CD45RO-
Effector memory CD8 T cells	CD3+ CD8+ CCR7- CD45RO+
Central memory CD8 T cells	CD3+ CD8+ CCR7+ CD45RO+
Naïve CD8 T cells	CD3+ CD8+ CCR7+ CD45RO-
Effector CD4 T cells	CD3+ CD4+ CCR7- CD45RO-
Effector memory CD4 T cells	CD3+ CD4+ CCR7- CD45RO+
Central memory CD4 T cells	CD3+ CD4+ CCR7+ CD45RO+
Naïve CD4 T cells	CD3+ CD4+ CCR7+ CD45RO-
Tregs	CD3+ CD4+ CD127- CD25+
T helper like cells (Th17)	CD3+ CD4+ CCR6+ CXCR3-
T helper like cells (Th2)	CD3+ CD4+ CCR6- CXCR3-
T helper like cells (Th1)	CD3+ CD4+ CCR6- CXCR3+
T follicular helper cells (Tfh)	CD3+ CD4+ CXCR5+
B cells	CD19+
Memory B cells	CD19+ $CD24^{hi}CD38^{-}$
Immature B cells	CD19+ $CD24^{hi}CD38^{hi}$
Atypical Memory B cells	CD19+ $CD24^{lo}CD38^{lo}$
Mature Naïve B cells	CD19+ $CD24^{int}CD38^{int}$
Plasmablasts	CD19+ $CD24^{-}CD38^{hi}$
Switched memory B cells	CD19+ CD27+ IgD-
Non-switched memory B cells	CD19+ CD27+ IgD+
Double negative (DN) B cells	CD19+ CD27- IgD-
DN CD11c-	CD19+ CD27- IgD- CD11c-
DN CD11c+	CD19+ CD27- IgD- CD11c+
DN1	CD19+ CD27- IgD- CD11c- CXCR5+
DN2	CD19+ CD27- IgD- CD11c+ CXCR5-
DN3	CD19+ CD27- IgD- CD11c- CXCR5-
DN4	CD19+ CD27- IgD- CD11c+ CXCR5+ IgE+
Naïve B cells	CD19+ CD27- IgD+
Myeloid cells	CD19- CD3-/CD66b-/CD56- HLA DR+
Non-classical Monocytes	CD19- CD3-/CD66b-/CD56- HLA DR+ CD14- CD16+
Intermediate Monocytes	CD19- CD3-/CD66b-/CD56- HLA DR+ CD14+ CD16+
Classical Monocytes	CD19- CD3-/CD66b-/CD56- HLA DR+ CD14+ CD16-
Plasmacytoid Dendritic cells	CD19- CD3-/CD66b-/CD56- HLA DR+ CD123+ BDCA2+

Table showing the marker combinations used to identify each cell population by flow cytometry. "hi", "lo", and "int" indicate high, low, and intermediate expression levels respectively.

Supplementary Table 2. JIA-uveitis patient demographics. Characteristics of JIA-uveitis patients split by disease activity into active and inactive groups.

Characteristic	Active uveitis	% M.D	Inactive uveitis	% M.D	P value (≤0.05 values shown)
**Total samples (n)**	29		15		
**Total patients (n)**	29		14		
**Age at sample, years, median, (range)**	6.7 (1.7 – 13.7)	0	11.3 (1.8 – 16)	0	
**Age at disease onset, years, median (range)**	2.7 (0.4 – 11.9)	0	2.8 (0.6 – 6.3)	0	
**Sex n (%)**					
**Female**	21 (72)	0	14 (93)	0	
**Male**	8 (28)	0	1 (7)	0	
**Ancestry n (%)**					
**Non-Caucasian**	11 (40)	0	4 (27)	0	
**Caucasian**	18 (60)	0	11 (73)	0	
**JIA subtype n (%)**					
**Oligoarticular – persistent**	12 (41)	0	3 (20)	0	0.029
**Oligoarticular – extended**	6 (21)	0	10 (67)	0	
**Polyarticular RF-ve**	9 (31)	0	2 (13)	0	
**Polyarticular RF+ve**	2 (7)	0	0 (0)	0	
**Enthesitis-related**	0 (0)	0	0 (0)	0	
**Psoriatic JIA**	0 (0)	0	0 (0)	0	
**Undifferentiated**	0 (0)	0	0 (0)	0	
**Active joint count, median, (range)**	4 (0 – 23)	3	1 (0 – 4)	0	
**ANA positive n (%)**	25 (86)	0	12 (80)	0	
**RF positive n (%)**	3 (10)	10	1 ()	0	
**HLA-B27 positive n (%)**	1 (3)	62	0 (0)	87	
**Treatment n (%)**					
**MTX**	6 (21)	0	10 (67)	0	0.007
**Anti-TNF**	0 (0)	0	2 (13)	0	
**Anti-IL6**	0 (0)	0	0 (0)	0	
**Topical eye steroids**	14 (48)	0	0 (0)	0	0.001
**Systemic steroids**	5 (17)	0	3 (20)	0	

Note: RF = Rheumatoid Factor, ANA = Anti-nuclear antibody, HLA-B27 = Human Leukocyte Antigen B27, MTX = Methotrexate. The significance of difference between groups was determined using a Chi-squared or Fishers exact test. Only p values ≤0.05 are shown and any variables that are not applicable to both groups are marked with N/A.

Supplementary Table 3. JIA-uveitis and future JIA-uveitis patient demographics. Characteristics of JIA-uveitis and JIA patients who go on to develop uveitis after sample date included within the study.

Characteristic	Future JIA-uveitis	% Missing data	JIA-uveitis	% Missing data	P value (≤0.05 values shown)
**Total samples (n)**	10		44		
**Total patients (n)**	10		43		
**Active uveitis n (%)**	N/A		29 (65)		N/A
**Recruitment years**	2007 - 2019		2010 - 2019		N/A
**Age at sample, years, median, (range)**	3.5 (1.8 – 11.9)	0	8.7 (1.7 – 16)	0	0.054
**Age at disease onset, years, median (range)**	2.4 (1.4 – 11.2)	0	4.3 (0.4 – 11.9)	0	
**Sex n (%)**					
**Female**	6 (60)	0	35 (79)	0	
**Male**	4 (40)	0	9 (21)	0	
**Ancestry n (%)**					
**Non-Caucasian**	1 (10)	0	15 (35)	0	
**Caucasian**	9 (90)	0	29 (65)	0	
**JIA subtype n (%)**					
**Oligoarticular – persistent**	1 (10)	0	15 (34)	0	
**Oligoarticular – extended**	3 (30)	0	16 (36)	0	
**Polyarticular RF-ve**	6 (60)	0	11 (25)	0	
**Polyarticular RF+ve**	0 (0)	0	2 (5)	0	
**Enthesitis-related**	0 (0)	0	0 (0)	0	
**Psoriatic JIA**	0 (0)	0	0 (0)	0	
**Undifferentiated**	0 (0)	0	0 (0)	0	
**'Polygo'**	10 (100)	0	42 (96)	0	
**Active joint count, median, (range)**	6 (1 - 8)	0	4 (0-23)	2	
**ANA positive n (%)**	10 (100)	0	37 (84)	0	
**RF positive n (%)**	1 (10)	7	4 (10)	7	
**HLA-B27 positive n (%)**	0 (0)	80	1 (8)	70	
**Treatment at time of sample n (%)**					
**MTX**	2 (20)	0	16 (36)	0	
**Anti-TNF**	0 (0)	0	2 (5)	1	
**Anti-IL6**	0 (0)	0	0 (0)	1	
**Topical eye steroids**	0 (0)	0	14 (32)	0	N/A
**Systemic steroids**	1 (10)	0	8 (18)	1	

Note: 'Polygo' = combined oligoarticular and polyarticular RF- JIA subtypes, RF = Rheumatoid Factor, ANA = Anti-nuclear antibody, HLA-B27 = Human Leukocyte Antigen B27, MTX = Methotrexate. The significance of difference between groups was determined using a Chi-squared or Fishers exact test. Only p values ≤0.05 are shown and any variables that are not applicable to both groups are marked with N/A.

Supplementary Table 4. JIA and JIA-uveitis patients with oligoarticular arthritis demographics. Characteristics of JIA and JIA-uveitis patients with oligoarticular arthritis.

Characteristic	JIA-uveitis	% Missing data	JIA	% Missing data	P value (≤0.05 values shown)
**Total samples (n)**	31		37		
**Total patients (n)**	30		37		
**Active uveitis n (%)**	18 (58)		N/A		N/A
**Recruitment years**	2010 - 2019		1999-2019		N/A
**Age at sample, years, median, (range)**	9.3 (1.7 – 16.0)	0	7.5 (2.4 – 16.6)	0	
**Age at disease onset, years, median (range)**	3.1 (0.6 – 11.7)	0	4.0 (0.2 – 16.0)		
**Sex n (%)**					
**Female**	26 (84)	0	27 (73)	0	
**Male**	5 (16)	0	10 (27)	0	
**Ancestry n (%)**					
**Non-Caucasian**	11 (36)	0	9 (24)	0	
**Caucasian**	20 (64)	0	28 (76)	0	
**JIA subtype n (%)**					
**Oligoarticular – persistent**	15 (48)	0	12 (32)	0	
**Oligoarticular – extended**	16 (52)	0	25 (68)	0	
**Active joint count, median, (range)**	2 (0 – 6)	1 (3)	3 (1 – 18)	1 (3)	
**ANA positive n (%)**	26 (84)	0 (0)	25 (71)	2 (5)	
**RF positive n (%)**	2 (7)	3 (10)	1 (3)	5 (14)	
**HLA-B27 positive n (%)**	0 (0)	21 (67)	0 (0)	31 (84)	
**Treatment at time of sample n (%)**					
**MTX**	11 (36)	0	13 (35)	0	
**Anti-TNF**	2 (7)	0	0 (0)	0	
**Anti-IL6**	0 (0)	0	0 (0)	0	
**Topical eye steroids**	10 (32)	0	N/A	0	N/A
**Systemic steroids**	6 (19)	0	1 (3)	0	0.04

Note: RF = Rheumatoid Factor, ANA = Anti-nuclear antibody, HLA-B27 = Human Leukocyte Antigen B27, MTX = Methotrexate. The significance of difference between groups was determined using a Chi-squared or Fishers exact test. Only p values ≤0.05 are shown and any variables that are not applicable to both groups are marked with N/A.

Supplementary Table 5. Aqueous humour sample patient demographics. Characteristics of JIA-uveitis patients included in the aqueous humour analysis.

Characteristic	JIA-Uveitis	% Missing data
**Total samples (n)**	2	0
**Total patients (n)**	2	
**Active uveitis n (%)**	100 (100)	
**Recruitment years**	2023 - 2024	
**Age at disease onset, years, median (range)**	6.5 (6.2 – 7.1)	0
**Age at sample, years, median (range)**	11 (9.1 -13.3)	
**Female**	0 (0)	0
**Male**	2 (50)	0
**Non-Caucasian**	1 (50)	0
**Caucasian**	1 (50)	0
**Oligoarticular – persistent**	1 (50)	0
**Oligoarticular – extended**	1 (50)	0
**ANA positive n (%)**	2 (100)	0
**HLA-B27 positive n (%)**	0 (100)	0
**Rheumatoid factor n (%)**	2 (100)	0
**Treatment at time of sample n (%)**		
**MTX**	1 (50)	0
**Anti-TNF**	1 (50)	0
**Anti-IL6**	1 (50)	0
**Systemic steroids**	0 (100)	0
**Topical eye steroids**	2 (100)	0

Note: ANA = Anti-nuclear antibody, MTX = Methotrexate, anti-TNF = tumour necrosis factor alpha antibody.

Supplementary Table 6. Enucleated eye patient demographics. Characteristics of historically biobanked JIA-uveitis patients included in the whole enucleated eye histological analysis.

**Characteristic**	JIA-Uveitis
**Total samples (n)**	3
**Total patients (n)**	3
**Active uveitis n (%)**	3 (100)
**Age at sample, median (range)**	32 (26 – 38)
**Female**	3 (100)
**Male**	0 (0)

Supplementary Table 7. List of fluorochrome-conjugated anti-human antibodies used for B cell/myeloid cell and T cell/NK cell immunophenotyping via conventional flow cytometry.

Antigen	Fluorochrome	Clone	Manufacturer	Final Dilution
**BDCA2**	PE	201A	Biolegend	1:100
**CD3**	FITC	OKT3	Biolegend	1:50
**CD56**	FITC	5.1H11	Biolegend	1:50
**CD66b**	FITC	G10F5	Biolegend	1:50
**CD16**	APC	3G8	Biolegend	1:50
**CD123**	PECY7	6H6	Biolegend	1:100
**HLA-DR**	PERCPCY5.5	L243	Biolegend	1:100
**CD24**	APC-CY7	ML5	Biolegend	1:50
**CD14**	AF700	63D3	Biolegend	1:100
**IgD**	BV421	IA6-2	Biolegend	1:50
**CD19**	BV510	HIB19	Biolegend	1:50
**CD38**	BV605	HIT2	Biolegend	1:50
**CD11c**	BV650	3.9	Biolegend	1:50
**CD27**	BV711	O323	Biolegend	1:50
**CCR7**	PE	G043H7	Biolegend	1:50
**TCRγδ**	FITC	11F2	BD biosciences	1:25
**CD25**	APC	BC96	Biolegend	1:50
**CD56**	PECY7	MEM-188	Biolegend	1:100
**CD45ro**	PERCPCY5.5	UCLH1	Biolegend	1:50
**CD4**	APC-CY7	OKT4	Biolegend	1:50
**CD8a**	AF700	SK1	Biolegend	1:100
**CXCR3**	BV421	G025H7	Biolegend	1:50
**CCR6**	BV510	G034E3	Biolegend	1:50
**CD3**	BV605	OKT3	Biolegend	1:50
**CXCR5**	BV650	RF8B2	BD biosciences	1:50
**CD127**	BV711	A019D5	Biolegend	1:50
**CD45ra**	PEDAZZLE	HI100	Biolegend	1:50

Supplementary Table 8. List of fluorochrome-conjugated anti-mouse antibodies used for general mouse immunophenotyping via conventional flow cytometry.

Antigen	Fluorochrome	Clone	Manufacturer	Final Dilution
**CD19**	BUV737	HIB19	BD Bioscience	1:100
**CD138**	BV711	281-2	BD Bioscience	1:200
**IgL**	FITC	RMK-45	Biolegend	1:100
**CD95**	PECY7	Jo2	Biolegend	1:200
**Blimp-1 (IC with eBioscience perm)**	APC	5e7	Biolegend	1:50
**GL7**	421/Pacific Blue	GL7	BD Bioscience	1:100
**IgD**	Percpcy5.5	11-26c.2a	Biolegend	1:100
**CD23**	BV711	B3B4	BD Bioscience	1:200
**IgM**	FITC	II/41	BD Bioscience	1:200
**CD21**	APC	7G6	Biolegend	1:100
**CD24**	421/Pacific Blue	M1/69	Biolegend	1:400
**CD19**	BV785	6D5	Biolegend	1:200
**CD4**	BV711	RM4-5	BD Bioscience	1:200
**CXCR5**	FITC	L138D7	Biolegend	1:100
**CD279**	PE	29F.1A12	Biolegend	1:200
**CD38**	PECY7	Rat IgG2a	Biolegend	1:200
**CD73**	APC	TY/11.8	Biolegend	1:100
**CD3**	FITC	17A2	Biolegend	1:200
**CD1d**	PerCyp5.5	1B1	BD Bioscience	1:200
**CD19**	BV421	6D5	Biolegend	1:100
**CD11b**	BV605	M1/70	Biolegend	1:200
**IgD**	BV786	11-26c.2a	BD Bioscience	1:200
**B220**	BUV395	RA3-6B2	BD Bioscience	1:200
**CD5**	BUV737	L17F12	BD Bioscience	1:200
**IgM**	APC CY7	RMM-1	Biolegend	1:200
**CD11c**	PeCy7	N418	Biolegend	1:200
**CD45**	BV510	30-F11	Biolegend	1:200

Supplementary Table 9. List of fluorochrome-conjugated anti-human antibodies used aqueous humour and whole blood immunophenotyping analysis via spectral flow cytometry.

Antigen	Fluorochrome	Clone	Manufacturer	Final Dilution
**CD45**	Alexa Fluor® 700	2D1	Biolegend	1:50
**CD19**	BD™ BUV615	HIB19	BD	1:25
**CD20**	Alexa Fluor® 488	2H7	Biolegend	1:50
**CD21**	RealBlue 780	B-ly4	BD	1:50
**CD24**	PerCP-eFluor710	eBioSN3 (SN3 A5-2H10)	Thermo	1:50
**CD27**	Brilliant Violet 650™	M-T271	BD	1:50
**CD38**	APC/Fire™ 810	HIT2	Biolegend	1:25
**IgD**	Brilliant Violet 480™	IA6-2	BD	1:50
**CD45RA**	PE-Dazzle 594	HI100	Biolegend	1:50
**CD3**	Spark Blue™ 550	SK7	Biolegend	1:50
**CD4**	Brilliant Violet 510™	SK3	BD	1:50
**CD8**	Brilliant Violet 570™	RPA-T8	Biolegend	1:50
**CD56**	BD™ BUV563	NCAM16.2	BD	1:50
**TCRγd**	BD™ BUV395	B1	BD	1:50
**CD25**	Brilliant™ Blue 700	M-A251	BD	1:50
**CD127**	PE/Fire™ 700	A019D5	Biolegend	1:50
**CXCR3**	PE	G025H7	Biolegend	1:50
**CCR6**	BD™ BUV661	11A9	BD	1:50
**CXCR5**	Brilliant Violet 421™	J252D4	Biolegend	1:50
**CCR7**	Brilliant Violet 785™	G043H7	Biolegend	1:50
**HLA-DR**	APC/Fire™ 750	L243	Biolegend	1:50
**CD16**	BD™ BUV496	3G8	BD	1:50
**CD14**	Spark Blue™ 574	HCD14	Biolegend	1:50
**CD123**	PerCP-Cy5.5	6H6	Biolegend	1:25
**iTCR/iNKT**	BD™ BUV805	6B11	BD	1:50
**Siglec 8**	Brilliant Violet 711™	837535	BD	1:50
**CD66b**	Alexa Fluor® 594	G10F5	Biolegend	1:50
**CD11c**	Brilliant Violet 605™	3.9	Biolegend	1:50
**BDCA2 (CD303)**	RealBlue 545	V24-785	BD	1:100
**CD279 (PD-1)**	PE/Cyanine7	EH12.1	BD	1:50
**CD86**	Alexa Fluor® 647	IT2.2	Biolegend	1:50
**CD69**	PE/Fire™ 640	FN50	Biolegend	1:50
**CD28**	Pacific Blue™	CD28.2	Biolegend	1:50

Supplementary Table 10. List of fluorochrome-conjugated anti-human antibodies used for cell sorting.

Antigen	Fluorochrome	Clone	Manufacturer	Final Dilution
**CD19**	PECY7	HIB19	Biolegend	1:25
