# Supplementary material for: Altered B cell activation contributes to the immunopathogenesis of childhood arthritis-associated uveitis
Source: Nat Commun. 2026 Feb 3;17:714. doi: 10.1038/s41467-025-68264-5 (PMC12868682; doi:10.1038/s41467-025-68264-5)
Supplement: Supplementary file 2 — Description of Additional Supplementary Files [file 41467_2025_68264_MOESM2_ESM.pdf]

Description of Additional Supplementary Files

Supplementary Data 1. Example bash script demonstrating how to convert MiXCR output into a format compatible with the Immcantation R package.

Supplementary Data 2. Schematic overview of the CLUSTER consortium data access workflow, illustrating step-by-step navigation from the European Genome-phenome Archive (EGA) study and dataset pages to the CLUSTER consortium website and the formal data access request process.
