# Supplementary material for: Altered B cell activation contributes to the immunopathogenesis of childhood arthritis-associated uveitis
Source: Nat Commun. 2026 Feb 3;17:714. doi: 10.1038/s41467-025-68264-5 (PMC12868682; doi:10.1038/s41467-025-68264-5)
Supplement: Supplementary file 3 — Supplementary Data 1 [file 41467_2025_68264_MOESM3_ESM.pdf]

```
#!/usr/bin/env bash
set -euo pipefail

# Requirements:
#   - MiXCR must be installed, and the mixcr executable must be on the
system PATH
#   - Immcantation must be installed, and the Immcantation scripts folder
must be on the system PATH
#   - NCBI IgBLAST must be installed (typically installed with
Immcantation), and the IgBLAST bin folder must be on the system PATH
#   - The IgBLAST database directory (typically installed with
Immcantation) must exist at the path defined in IGBLAST_DB
#   - The IMGT germline directory (typically installed with Immcantation)
must exist at the path defined in GERMLINE_DIR


# 1) User configuration

# Input paired-end FASTQ files (forward and reverse read files, often R1
and R2)
FORWARD_READ="/path/to/forward.fastq.gz"
REVERSE_READ="/path/to/reverse.fastq.gz"

# Immcantation resource directories
IGBLAST_DB="/path/to/immcantation/share/igblast"
GERMLINE_DIR="/path/to/immcantation/share/germlines/imgt/human/vdj"

# Define sample name and make output directory
SAMPLE="sample1"
OUT_DIR="/path/to/output/${SAMPLE}"
mkdir -p "$OUT_DIR"


# 2) Extract B-cell receptor sequences from bulk RNA-seq using MiXCR

mixcr analyze rna-seq --species hsa "$FORWARD_READ" "$REVERSE_READ"
"$OUT_DIR/$SAMPLE"


# 3) Convert MiXCR clones table to FASTA

# Define variables for the files involved (check MiXCR naming convention)
TSV="$OUT_DIR/${SAMPLE}.clones_IGH.tsv"
FASTA="$OUT_DIR/${SAMPLE}.fasta"

# MiXCR's clones_IGH.tsv contains one row per clone.
# Relevant columns:
#   column 1 = cloneId (unique identifier for each clone)
#   column 2 = cloneCount (read depth / clone abundance)
#   column 4 = CDR3 nucleotide sequences (one or more, separated by
commas)
#
# The awk command:
#   - splits the column 4 CDR3 field into individual sequences,
#   - selects the longest CDR3 sequence for that clone,
#   - writes a FASTA record with header:
#         >sample_cloneId_cloneCount
```

```
#     so that clone identity and clone abundance are preserved for
downstream analysis.
awk -v NAME="$SAMPLE" -F'\t' 'NR>1 {
  n = split($4, seqs, ",")
  longest = ""
  for (i=1; i<=n; i++) {
    if (length(seqs[i]) > length(longest)) {
      longest = seqs[i]
    }
  }
  print ">" NAME "_" $1 "_" $2
  print longest
}' "$TSV" > "$FASTA"


# 4) Run standard Immcantation steps: assigngenes, MakeDB,
CreateGermlines

# Define variables for the files involved (check IgBLAST naming
convention)
FMT7="$OUT_DIR/${SAMPLE}_igblast.fmt7"
DBPASS_TSV="$OUT_DIR/${SAMPLE}_igblast_db-pass.tsv"

assigngenes.py igblast -s "$FASTA" -b "$IGBLAST_DB" --organism human --
loci ig --format blast

MakeDB.py igblast -i "$FMT7" -s "$FASTA" -r "$GERMLINE_DIR"/* --extended
--partial

CreateGermlines.py -d "$DBPASS_TSV" -g dmask -r
"$GERMLINE_DIR/imgt_human_IGHV.fasta"
"$GERMLINE_DIR/imgt_human_IGHD.fasta"
"$GERMLINE_DIR/imgt_human_IGHJ.fasta"
```
