# Supplementary material for: Altered B cell activation contributes to the immunopathogenesis of childhood arthritis-associated uveitis
Source: Nat Commun. 2026 Feb 3;17:714. doi: 10.1038/s41467-025-68264-5 (PMC12868682; doi:10.1038/s41467-025-68264-5)
Supplement: Supplementary file 4 — Supplementary Data 2 [file 41467_2025_68264_MOESM4_ESM.pdf]

DATA ACCESS STEP BY STEP

Manuscript title: Altered B and T cell interactions contribute to the immunopathogenesis of childhood arthritis-associated uveitis.
Date created: 09/12/2025
EGA study hyperlink: https://ega-archive.org/studies/EGAS50000001123
EGA dataset hyperlink: https://ega-archive.org/datasets/EGAD50000001616

EGA STUDY WEBPAGE

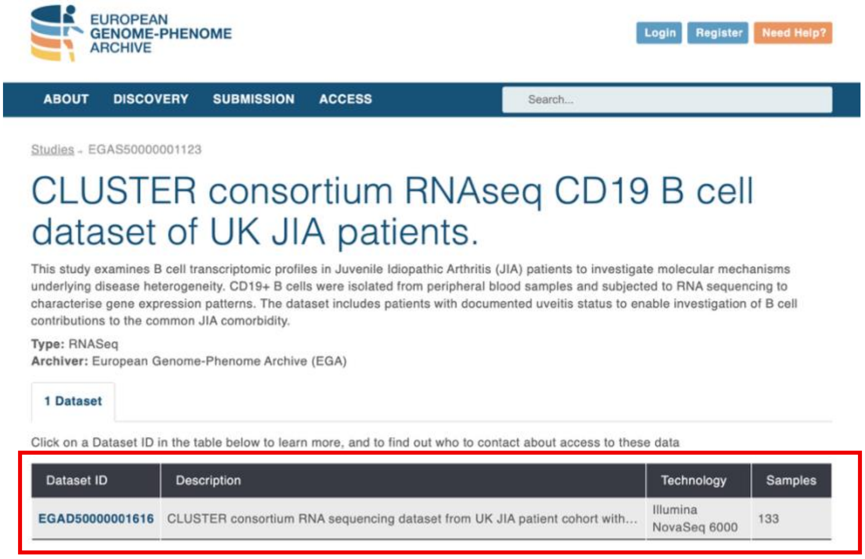

STEP 1: Click through to dataset (red box)
EGA DATASET WEBPAGE

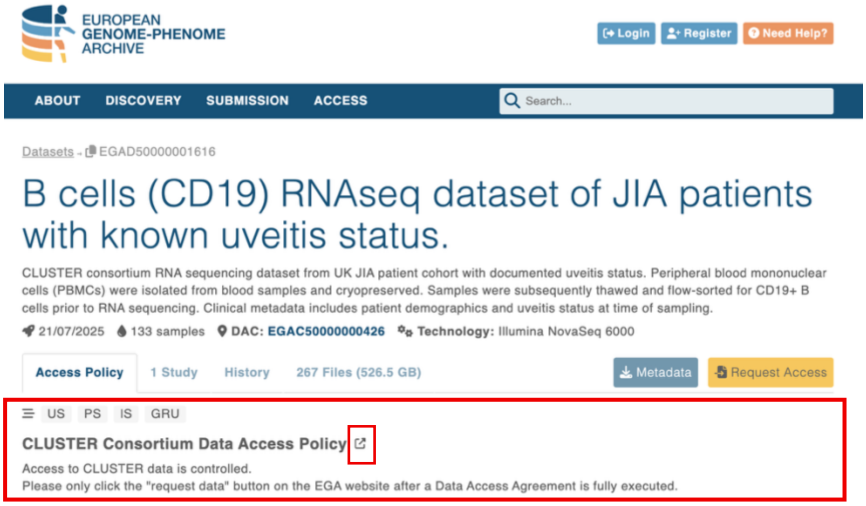

STEP 2: Click on the ‘CLUSTER consortium Data Access Policy’ to take you through to the CLUSTER wesbite where you can read about the data access policy.

Next page….
CLUSTER website data access page

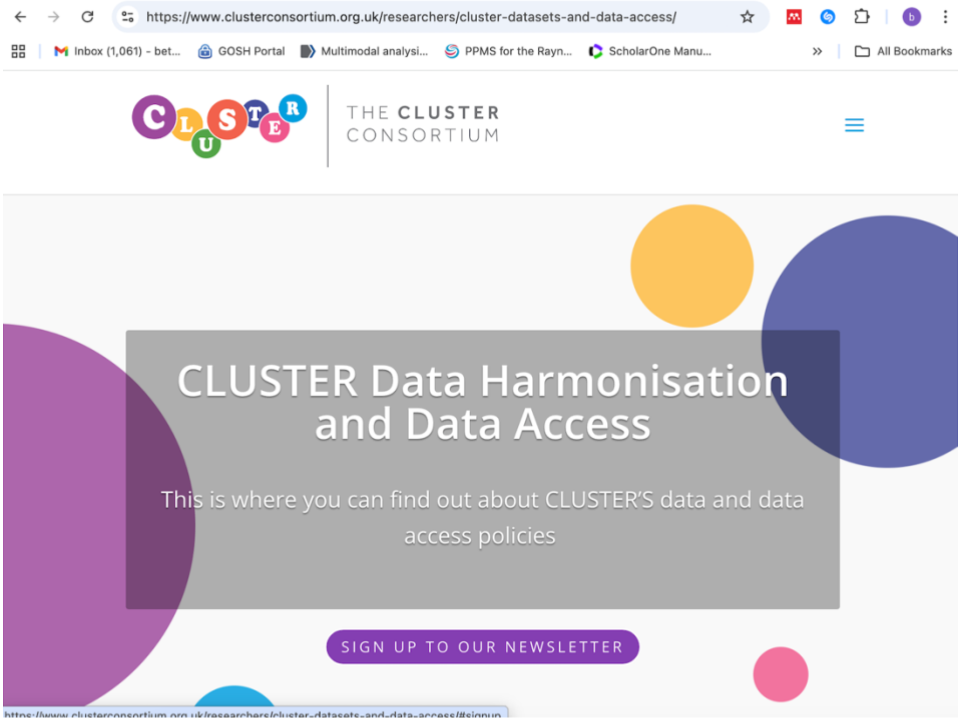

STEP 3: Scroll down to see the CLUSTER datasets section and the Zenodo link.

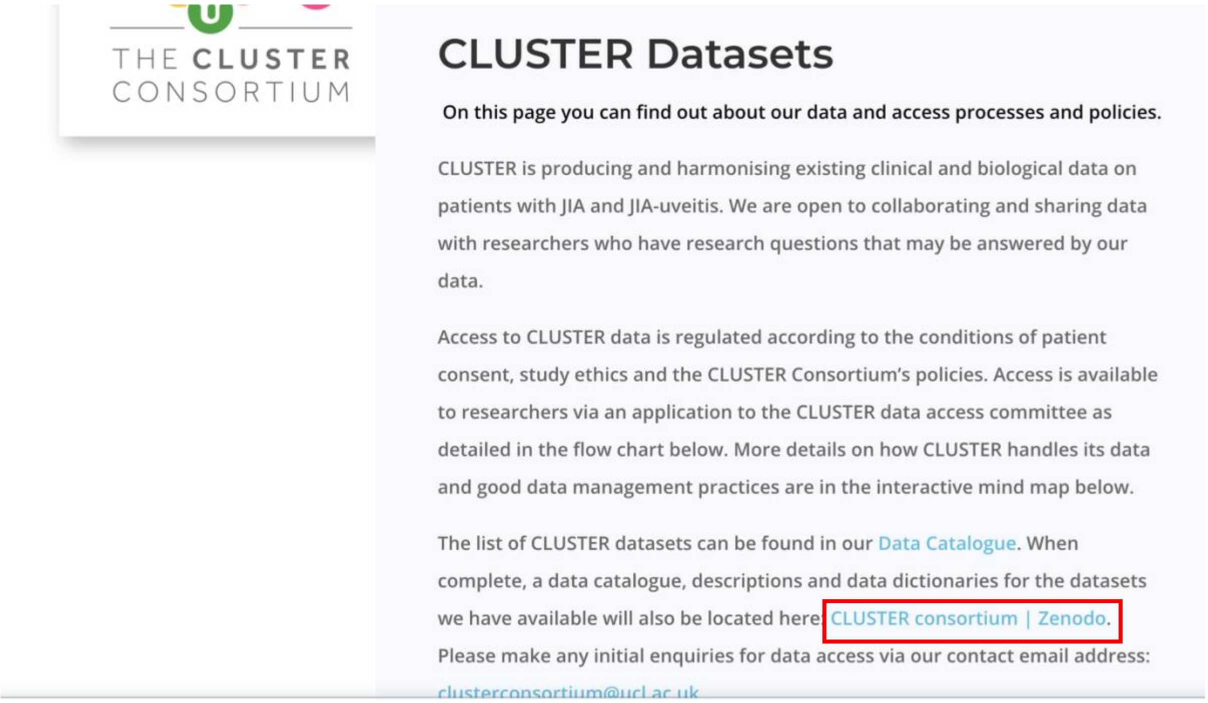

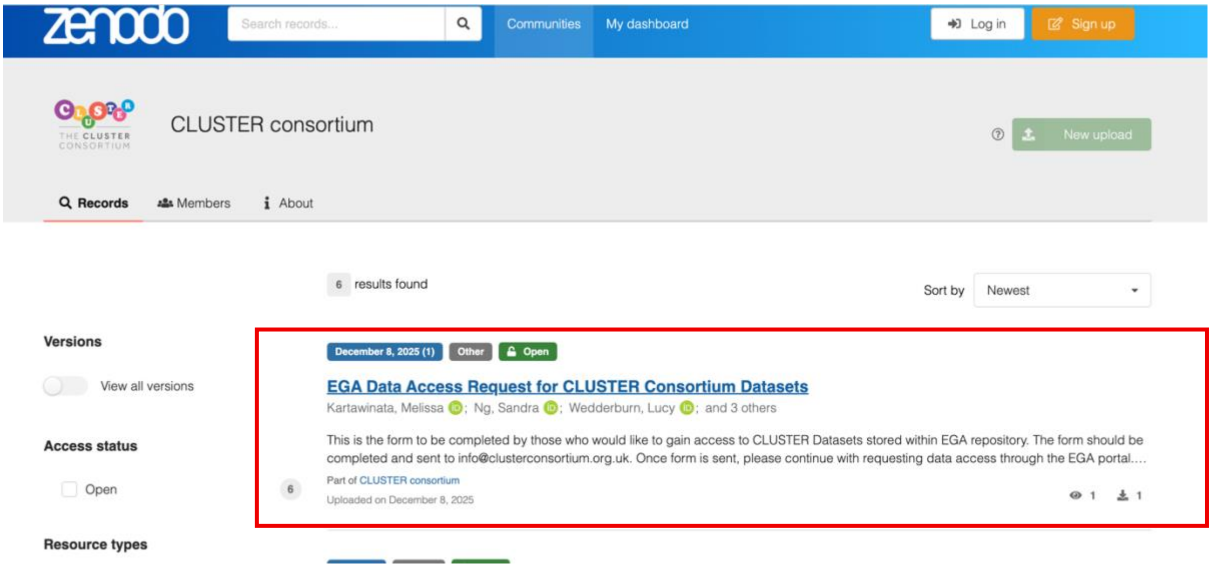

STEP 4: Click on ‘EGA Data Access Request for CLUSTER Consortium Datasets’.

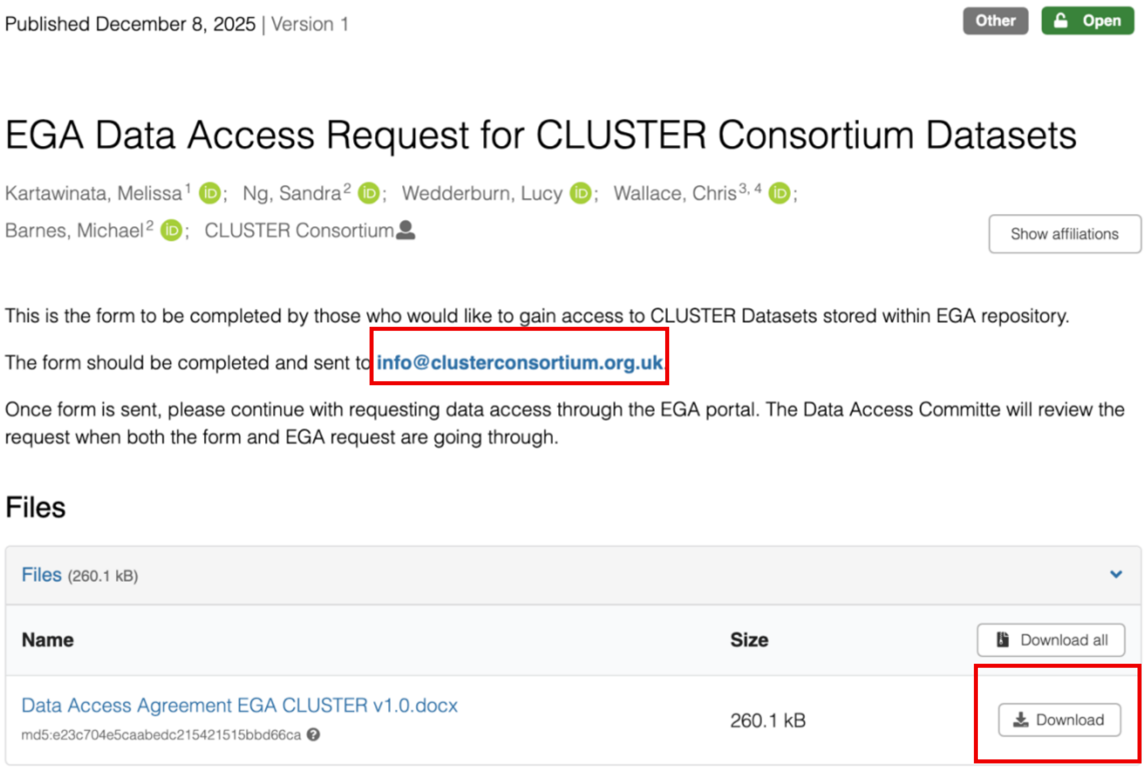

STEP 5: Download this form, fill it in, and return to info@clusterconsortium.org.uk.

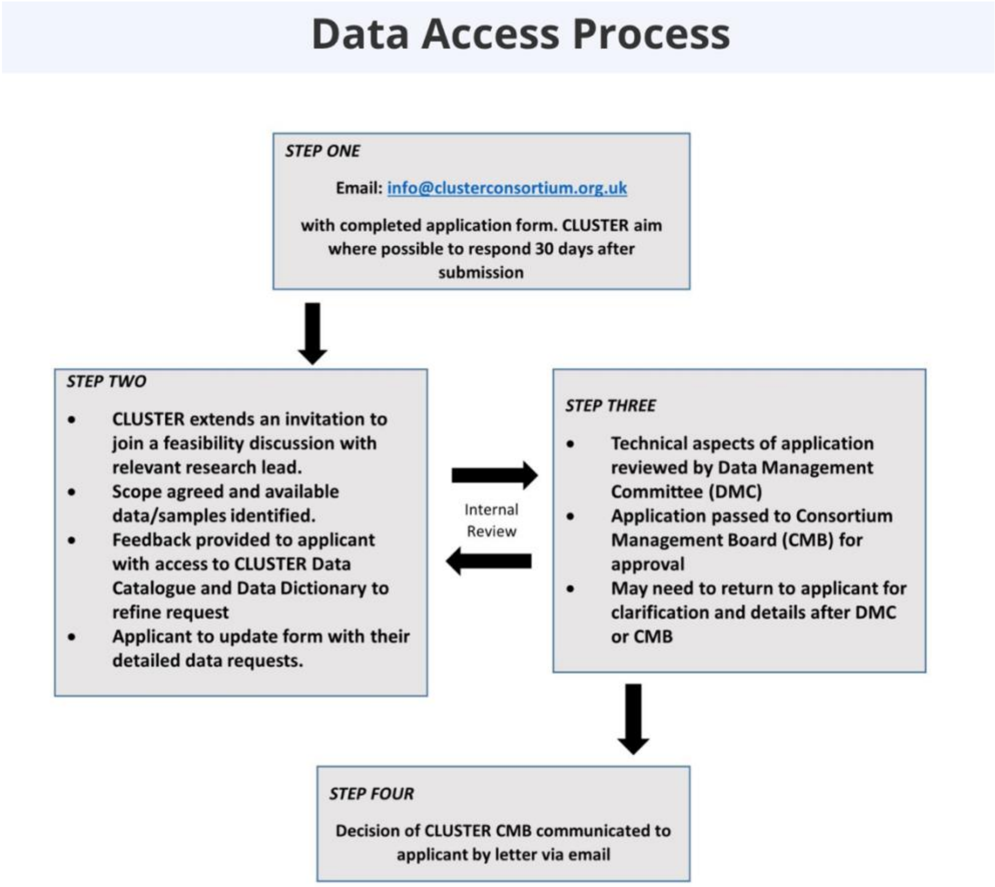

This is an overview schematic of the CLUSTER consortium data access workflow.
